# An Integrated Approach for Platoon-based Simulation and Its Feasibility Assessment

**DOI:** 10.1371/journal.pone.0114406

**Published:** 2015-03-18

**Authors:** Kok Mun Ng, Mamun Bin Ibne Reaz

**Affiliations:** 1 Faculty of Electrical Engineering, Universiti Teknologi Mara, Shah Alam, Selangor, Malaysia; 2 Department of Electrical, Electronic and Systems Engineering, Faculty of Engineering and Built Environment, Universiti Kebangsaan Malaysia, UKM Bangi, Selangor, Malaysia; Tianjin University of Technology, CHINA

## Abstract

Research on developing mathematical and simulative models to evaluate performance of signalized arterials is still ongoing. In this paper, an integrated model (IM) based on Rakha vehicle dynamics and LWR model is proposed. The IM which imitates actuated performance measurement in signalized arterials is described using continuous timed Petri net with variable speeds (VCPN). This enables systematic discretized description of platoon movement from an upstream signalized intersection towards a downstream signalized intersection. The integration is based on the notion that speed and travel time characteristics in a link can be provided using Rakha model. This will assist the LWR to estimate arrival profiles of vehicles at downstream intersection. One immediate benefit of the model is that platoon arrival profile obtained from the IM can be directly manipulated to estimate queues and delays at the target intersection using input-output analysis without considering the effect of shockwaves. This is less tedious as compared to analysing the LWR model through tracing trajectory of shockwave. Besides, time parameters of a platoon could be estimated for self-scheduling control approach from a cycle to cycle basis. The proposed IM is applied to a test intersection where simulated queues and average delays from the IM are compared with the platoon dispersion model (PDM) implemented in TRANSYT, cell transmission model (CTM) and HCM2000 for both under-saturated and oversaturated situations. The comparisons yielded acceptable and reasonable results, thus ascertained the feasibility and validity of the model.

## Introduction

The problem of traffic congestions in urban networks is a major concern in many countries. Throughout these few decades, much effort had been poured in to develop traffic evaluation models and control strategies that can help ease traffic congestions. One aspect of traffic research that is still ongoing is related to coordinated arterials which involve movement of a platoon or platoons of vehicles. The research on coordinated arterials includes (i) modeling platoon progression to evaluate performance of signalized arterials e.g. queues and delays [1–4]; (ii) developing real-time traffic control strategies to optimize platoon progression in coordinated arterials [5–6] and (iii) analyzing traffic flow in complex traffic network [7].

In real-time performance estimation, the movement of cohesive vehicles in arterial can be captured using road detectors. One detector is placed at the stop line and another at a fixed distance upstream on the road [8]. The arrival profile of incoming vehicles is tracked by periodically sampling the upstream sensor. Departing vehicles at the stop line are monitored via the stop line detector. Performance of the signalized intersection such as queues and delays can be estimated using input-output technique based on the profiles of arrived and departed vehicles [8–9]. Recently, an improved virtual probe model for estimating arterial travel time and queue lengths under congested conditions has been proposed due to the limitation in the input-output method to estimate queue and arterial travel time in congested links [10]. These works indicate some of the real-time estimation methods available to estimate performances of signalized arterials.

The objective of this research is to propose a new platoon-based simulative model that could “imitate” the operation of real-time performance measurement in [8] and [9]. In this platoon-based model, we proposed integration of the existing Lighthill, Witham and Richards (LWR) model [11–12] with Rakha vehicle dynamics model [13] to predict platoon movement and arrivals in signalized arterials. This integrated model (IM) demonstrated that the arrival profile obtained could be analyzed directly using input-output analysis to estimate queues and delays from a cycle to cycle basis at signalized arterials without considering the effect of shockwave. This notion is attributed to Newell [14] and is found to be less tedious compared to tracing the trajectory of shockwaves and evaluation of shock path [15–16]. Another benefit of the model is the ability to provide time parameters of platoons which may be useful for self-scheduling approach [5–6].

A continuous timed Petri net with variable speeds (VCPN) [17–18] is adopted to describe the LWR. The LWR described via VCPN could provide a more “relaxed” and systematic representation of continuous timed models [19]. Instead of referring to a fundamental diagram as in shock path analysis, Rakha model is integrated to provide speed and travel time characteristics of vehicles. These important characteristics provide vehicles arrival information which is useful for the LWR-VCPN model.

In a previous paper, the IM has been proven capable of producing arrival profiles of platoons and predicting platoon size (clearance time) and travel time accurately [20]. However, the IM needs further enhancement for it to be implemented to predict performances in an actual arterial. In addition, a comparative assessment with other traffic models needs to be done to evaluate the feasibility of the IM. In this paper, the authors (i) develop and explain further enhancement of the IM; (ii) conduct a comparison of the feasibility of the IM to estimate maximum end of red queue (Q_MEOR_), mean maximum queue (Q_MM_) and average delays with platoon dispersion model (PDM) in TRANSYT [2], the cell transmission model (CTM) by Daganzo [21] and the Highway Capacity Manual 2000 (HCM2000) [22]. A test intersection is modelled by the respective models. Queues and average delays simulated by IM are compared to TRANSYT, CTM and HCM2000 computations for both under-saturated and oversaturated traffics to ascertain the model feasibility.

Section 2 provides a brief overview of the PDM, CTM and the HCM2000 methodologies. In Section 3 of this paper, the theoretical background of vehicle dynamics model, particularly Rakha vehicle dynamics model and the LWR theory are briefly explained. The proposed IM which derived its existence from these two models and its underlying methodology is explained and justified in section 4. Section 5 describes the IM application and implementation into a test intersection with fixed-time control. Section 6 presents simulation results and evaluation of the model feasibility in simulating Q_MEOR_, Q_MM_ and average delays via comparison of model-predicted results with results from TRANSYT, CTM and HCM2000. Concluding remarks are given in section 7.

## A Brief Review on Existing Models

### 2.1 Platoon Dispersion Model (PDM)

The well-known model of platoon dispersion is developed by Robertson [2]. It has been successfully implemented in the TRANSYT simulation and optimization software. The model is derived by assuming a geometric distribution of vehicles travel time as depicted in equation (1) where *q*_2_(*i*) is the number of vehicles passing the downstream observation point; *q*_1_(*i*) is the number of vehicles released from upstream signal; *t* is the average travel time; *i* is the time step and α is the platoon dispersion factor.


$$q_{2}\left(i+t\right)=\frac{1}{1+\alpha t}q_{1}\left(i\right)+\left(1-\frac{1}{1+\alpha t}\right)q_{2}\left(i+t+1\right)$$ (1)

A flow histogram of vehicles leaving the upstream stop line is first constructed. The flow histogram is transformed by equation (1) to obtain the arrival profile at downstream stop line. The rate of platoon dispersion depends on the value of α. Robertson’s model has shown satisfactory agreement with field data under under-saturated flow condition.

### 2.2 Cell Transmission Model (CTM)

The cell transmission model [21] simulates traffic flow over a one-way road without any intermediate entrances or exits. Vehicles enter at one end and leave at the other. It is considered as a discrete approximation of the hydrodynamic model with a density-flow relationship in the shape of a trapezoid. The road is divided into homogeneous sections called *cells*, numbered from *i* = 1 to I. The road system which comprised of a series of cells is implemented with a “time-scan” strategy where the advancement of vehicles from one cell to the next is updated with every tick of a clock. The lengths of the cells are set equal to the distances travelled in light traffic by a typical vehicle in one clock tick.

The flow advancement equation shown in equation (2) can be written as the cell occupancy at time *t* + 1 equals its occupancy at time *t*, plus the inflow and minus the outflow where *n_i_*(*t* + 1) is the cell occupancy at time *t* + 1; *n_i_*(*t*) is the cell occupancy at time *t*; *y_i_*(*t*) is the inflow at time *t* and *y*_*i* + 1_(*t*) is the outflow at time *t*.


$$n_{i}\left(t+1\right)=n_{i}\left(t\right)+y_{i}\left(t\right)-y_{i+1}\left(t\right)$$ (2)

The flows are related to the current conditions at time *t* as indicated in equation (3). *N_i_*(*t*) is the maximum number of vehicles that can be present in cell *i* at time *t*; *n*_*i*−1_(*t*) is the number of vehicles in cell *i* – 1 at time *t*; *Q_i_*(*t*) is the capacity flow into cell *i* for time interval *t* and {*N_i_*(*t*)−*n_i_*(*t*)} is the amount of empty space in cell *i* at time *t*.


$$y_{i}\left(t\right)=\text{min}\left[n_{i-1}\left(t\right),Q_{i}\left(t\right),\alpha \left\{N_{i}\left(t\right)-n_{i}\left(t\right)\right\}\right]$$ (3)

$$\alpha =\{\begin{array}{cc} 1 & n_{i-1}\left(t\right)\leq Q_{i}\left(t\right)\\ w/v & n_{i-1}\left(t\right)>Q_{i}\left(t\right) \end{array}$$ (4)

The amount of empty space in cell *i* is multiplied with α as depicted in equation (4) where *v* is the free flow speed of the vehicles and *w* is the speed of backward wave. A value of *α* = *w/v;* is applicable when the number of vehicles in cell *i*−1 exceeds the capacity flow in cell *i* (i.e. *n*_*i*−1_(*t*)>*Q_i_*(*t*)). Hence, equation (3) allows backward waves with speed *w* ≤ *v*. This is a realistic model as backward wave speed (*w*) will not always be the same as free flow speed (*v*). The speed of the backward wave can be approximated from the flow-density diagram.

### 2.3 HCM 2000 Model (HCM2000)

In the HCM2000 [22], the average delay per vehicle for a lane group is given by equation (5) where *d* is the average overall delay per vehicle; *d*_1_ is the uniform delay; *d*_2_ is denoted as incremental delay; *d*_3_ represents the residual demand delay to account for over-saturation queues that may have existed before the analysis period and *PF* depicts the adjustment factor for the effect of the quality of progression in coordinated systems and is applied to the uniform delay. However, the *PF* is not applied at oversaturated conditions when there is an initial queue.


$$d=d_{1}\left(PF\right)+d_{2}+d_{3}$$ (5)

The following equations further detailed the calculations of the uniform and incremental delay components shown in (6) and (7):

$$d_{1}=\frac{0.5C\left(1-u\right)}{\left(1-u\;\text{min}\left(X,1.0\right)\right)}$$ (6)

$$d_{2}=900T\;\left[\left(X-1\right)+\sqrt{{\left(X-1\right)}^{2}+\frac{8kIX}{cT}}\right]$$ (7)

*X* is the degree of saturation (DOS) for lane group; *C* is the cycle length (s); *u* is the ratio of effective green time to cycle length; *T* is the duration of analysis period (h); *c* depicts the lane group capacity (veh/h); *k* represents the incremental delay adjustment for the actuated control and *I* is the incremental delay adjustment for filtering or metering by upstream signal.

The progression factor (*PF*), which is a function of *P* is the proportion of all vehicles arriving during the green period and can be calculated using equation (8) where *f_PA_* is the supplemental adjustment factor for platoon arrival during the green period. Values of *P* and *f_PA_* can be calculated using default values provided by HCM2000.

$$PF=\frac{\left(1-P\right)f_{PA}}{\left(1-u\right)}$$ (8)

The initial queue delay *d*_3_ is estimated when there is an initial queue during the analysis period. If the traffic is under-saturated or there is no initial queue at the start of the analysis period, *d*_3_ = 0.

The HCM2000 computation for the mean maximum queue (average back of queue) is given by equation (9). Equation (9) comprises of two terms *Q*_1_ and *Q*_2_. The first term represents non-random (uniform) back of queue and all randomness and oversaturation effect are accounted in the second term.


$$Q=Q_{1}+Q_{2}$$ (9)

$$Q_{1}=PF_{2}\frac{VC\left(1-u\right)}{1-\left[u\;\text{min}\left(X,1.0\right)\right]}$$ (9.1)

$$Q_{2}=0.25cT\;\left[\left(X-1\right)+\sqrt{{\left(X-1\right)}^{2}+\frac{8k_{B}X}{cT}}\right]$$ (9.2)

$$PF_{2}=\frac{\left(1-P\right)\left(1-V/s\right)}{\left(1-u\right)\left(1-R_{p}\left(V/s\right)\right)}$$ (10)

$$k_{B}=0.12{\left(sg\right)}^{0.7}I$$ (11)

Most of the notations in equation (9), (9.1), (9.2), (10) and (11) are similar to those used to calculate delays in equation (6) and (7). Additional notations used in these equations are *V* which is the demand flow rate (veh/h); *s* is the saturation flow rate (veh/h); *g* is the effective green time; *k_B_* is an adjustment factor used for pre-timed signals and $R_{p}=\frac{P}{u}$ is the platoon ratio.

## Theoretical Background

### 3.1 Rakha Vehicle Dynamics Model

Vehicle acceleration in particular has a significant impact on several factors in traffic engineering. These include the analysis of signalized intersections, traffic modeling, and the design of roadway characteristics. The acceleration capability of a vehicle is also an important factor in the investigation of certain road accidents. The rest of this section is dedicated to explain the constant power vehicle dynamics model [13]. The constant power vehicle dynamics model by Rakha et al. [13] is based on the basic principle of physics that force equals mass times acceleration. If the net force *F* on the vehicle and the vehicle mass *M* are known, the acceleration of the vehicle can be determined by equation (12). The net force on the vehicle is the difference between the tractive force *F* applied by the vehicle and the various resistance forces *R*, the vehicle encounters as it travels. The mass *M* of the vehicle is constant, but the magnitude of the applied force and the resistance forces are variables.


$$a=\frac{F-R}{M}$$ (12)

The net force is defined in (13) as a minimum function of the varying tractive force *F_t_* and the maximum tractive force attainable *F_max_*. The tractive force *F_t_* in (14) is a function of the ratio between the vehicle speed *v* and the engine power *P* multiplied with the power transmission efficiency η. However, the maximum attainable tractive force *F_max_* is constrained by the friction between the vehicle tires on the tractive axle and the roadway pavement else higher tractive forces would result in wheel spin. This maximum tractive force (15) is a function of the mass of the vehicle on the tractive axle *M_ta_* and the coefficient of friction *μ* between the tires and the pavement.


$$F=\text{min}\left(F_{t},F_{\text{max}}\right)$$ (13)

$$F_{t}=3600\eta \frac{P}{v}$$ (14)

$$F_{\text{max}}=9.8066M_{ta}\mu$$ (15)

Total resistance force *R* is the next variable of interest in (12). Three major types of resistance forces need to be considered namely aerodynamic resistance *R_a_*, rolling resistance *R_r_*, and grade resistance *R_g_*. The total resistance force is simply computed as the sum of these three resistance components, as shown in (16). For further details on the calculations of *R_a_*, *R_r_*, and *R_g_*; the reader can refer to [13].


$$R=R_{a}+R_{r}+R_{g}$$ (16)

Therefore, given that acceleration is the second derivative of distance with respect to time, (12) resolves to a second-order ordinary differential equation (ODE) function of the form indicated in (17).


$$x=f\left(\dot{x},x\right)$$ (17)

Equation in (12) can be rewritten as a second-order ODE as shown in (18) where *F*(*t_i_*) is the net force at instance *t_i_*; *R*(*t_i_*) is the total resistance force at instance *t_i_* and *a*(*t_i_*) is the vehicle acceleration at instance *t_i_*. The relationship in (18) can be further recast as a system of two first-order equations as presented in (19). These ODEs are further solved using first-order Euler approximations as demonstrated in (20) and (21) where *t_i_* = *t_0_* + *i*.Δt for *i* = 1,2,..,n; Δt depicts the duration of interval used to solve the ODE; *v*(*t_i_*) is vehicle speed at instance *t_i_*; and *x*(*t_i_*) is vehicle location along test section at instance *t_i_*. The acceleration at *i* = 1 is calculated using equation (18) whereas the speed and the location of the vehicle are calculated using equation (20) and (21) respectively using the acceleration at *i* = 0 (*t* = *t_0_*).


$$a\left(t_{i}\right)=\frac{F\left(t_{i}\right)-R\left(t_{i}\right)}{M}$$ (18)

$$\left\{\begin{array}{c} \dot{v}\left(t_{i}\right)\\ \dot{x}\left(t_{i}\right) \end{array}\right\}=\left\{\begin{array}{c} a\left(t_{i}\right)\\ v\left(t_{i}\right) \end{array}\right\}$$ (19)

$$v\left(t_{i}\right)=v\left(t_{i-1}\right)+a\left(t_{i-1}\right)\Delta t$$ (20)

$$x\left(t_{i}\right)=x\left(t_{i-1}\right)+v\left(t_{i-1}\right)\Delta t$$ (21)

With derivation of (18), (20) and (21), the acceleration, speed and location of a particular vehicle starting from the stop line of an upstream signal towards a downstream intersection can be easily simulated. The trajectory of the lead vehicle and the speed of the vehicle determine values of flow *q*(*x,t*) when density *ρ*(*x,t*) along the trajectory is known based on the definition in equation (23).

### 3.2 The LWR based VCPN Model

The rest of this section outlines the theory of the LWR model and how it could be described using the VCPN by Tolba et al. [17–18]. The LWR model is a macroscopic model that describes traffic flow similar to fluid flows. It comprises of three basic postulates namely the conservation law (22); the continuous timed relationship between traffic flow, density and speed (23); and the fundamental diagram (24). The traffic density is denoted by *ρ*(*x,t*); *q*(*x,t*) denotes the traffic flow rate; and traffic speed is represented by *S*(*x,t*).


$$\frac{\partial \rho \left(x,t\right)}{\partial t}+\frac{\partial q\left(x,t\right)}{\partial t}=0$$ (22)

q(x,t)=ρ(x,t).S(x,t) (23)

q(x,t)=f(ρ(x,t)) (24)

In practice, the model is usually discretized in both time and space. The discretization in time is done by considering time steps whereas the discretization in space is obtained by dividing the motorway into sections of different lengths. In Tolba et al. [17–18] a motorway section without any on-ramp or off-ramp can be divided into segments (see Fig 5 in [17]). Each segment has its respective density *ρ*(*t*), flow rate *q*(*t*) and traffic speed *S*(*t*) that varies with respect to time and relate with each other according to the definitions in (22)–(24).

Tolba et al. [17–18] illustrated the description of the LWR model by mean of a VCPN (refer to Fig 4 in [17]). The basic VCPN shown in Fig 4 in [17] describes section *i* of the motorway with length of *Δi*. Each place *P_i_* corresponds to the road segment *i* with *i* = 1, …, L whereas transition *T_i_* stands for the separation between the segments *P_i_* and *P_i+1_*. The marking *m_i_*(*t*) of place *P_i_* represents the number of vehicles in the considered segment and the transition firing speed *v_i_*(*t*) stands for the average flow rate *q_i_*(*t*). Hence, the average flow density *ρ_i_*(*t*) and average speed *S_i_*(*t*) for segment *i* are given by (25) and (26) respectively.


$$\rho_{i}\left(t\right)=\frac{m_{i}\left(t\right)}{\Delta_{i}}$$ (25)

$$S_{i}\left(t\right)=\frac{v_{i}\left(t\right).\Delta_{i}}{m_{i}\left(t\right)}$$ (26)

Both (25) and (26) satisfy the continuous representation of the LWR theory as defined in (23). On the other hand by inspection of (22), the conservation of the number of vehicles in segment *i* is given by (27) whereas (28) can be derived when (25) is differentiated.

$$\frac{d\rho_{i}\left(t\right)}{dt}+\frac{q_{i}\left(t\right)-q_{i-1}\left(t\right)}{\Delta_{i}}=0$$ (27)

$$\frac{d\rho_{i}\left(t\right)}{dt}=\frac{1}{\Delta_{i}}.\frac{dm_{i}\left(t\right)}{dt}$$ (28)

By substituting (28) into (27), we obtained,

$$\frac{dm_{i}\left(t\right)}{dt}=v_{i-1}\left(t\right)-v_{i}\left(t\right)$$ (29)

Equation (29) corresponds to the marking evolution of the place *P_i_* in the VCPN model. The transition firing speed is a piecewise linear function of the marking. In the VCPN model in [17] (i.e. Fig 4 in [17]), the additional place $P_{i+1}^{\;'}$ corresponds to the number of available sites in the downstream segment *i + 1*. The firing speed *v_i_(t)* of the transition *T_i_* is defined as (30). It depends on the marking of the place *P_i_* (i.e., on the number of the vehicles in the upstream segment *i*); the marking of the place $P_{i+1}^{\;'}$ (i.e., the number of sites available in the downstream segment *i + 1*) as well as *α_i_(t)* which functions to limit the number of simultaneous firing of *T_i_*.


$$v\left(t\right)=v_{\text{max}\;i}\;\text{min}\left(m_{i}(t\right),m_{i+1}^{'}\left(t\right),\alpha_{i}\left(t\right))$$ (30)

The maximal firing frequency *v*_max *i*_ of transition *T_i_* (a constant value) is defined in (31). It is dependent on the limited maximal speed *v*_*free i*_ in segment *i*.


$$v_{\text{max}\;i}=\frac{v_{\mathrm{free}\;i}}{\Delta_{i}}$$ (31)

This VCPN model was successfully implemented on a motorway to estimate flow rate, average speed and fundamental diagram in [18]. We have found the VCPN to be well suited for the description of the LWR model as it provides a more “relaxed” model as compared to a purely discrete model which can leads to large net and the state explosion problem [19]. Based on synthetic speeds from the Rakha model, flows along different segments of the motorway or arterial could be determined. Thus, one can construct a cumulative arrival profile at any point along the trajectory travelled by the platoon. Subsequently, the profile obtained assists in queues and delays estimation.

## Proposed Integrated Model and Analysis Method

**Fig 1:**
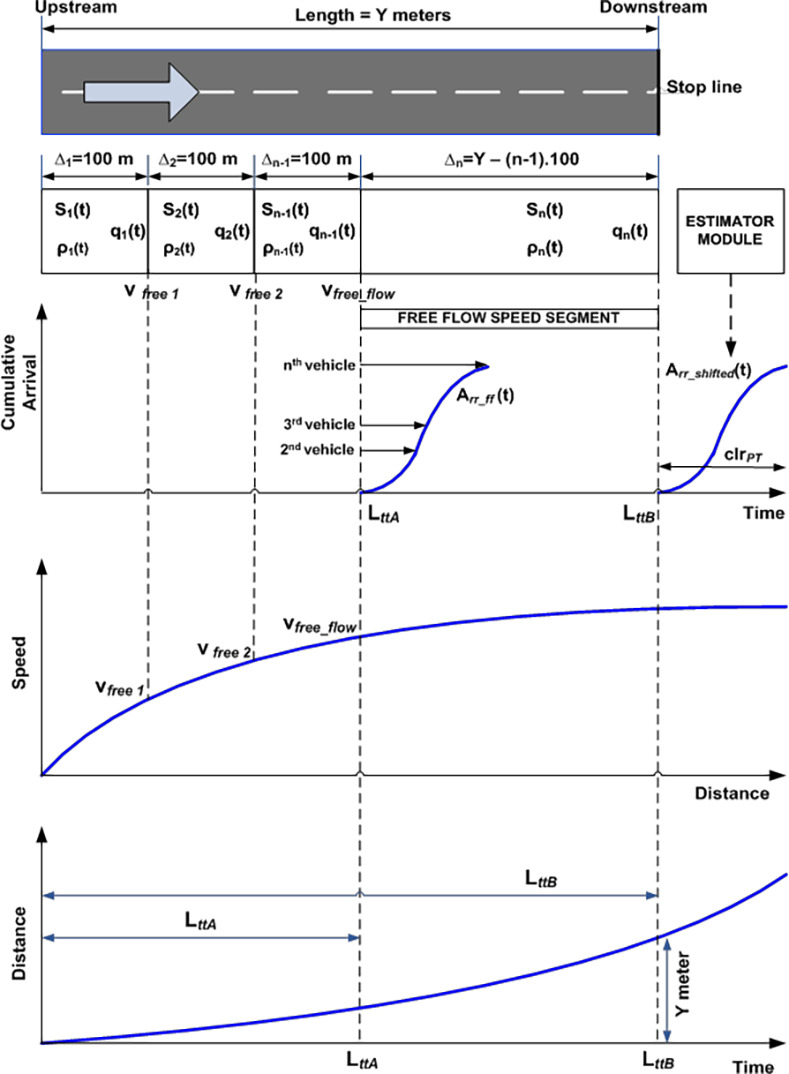
Integrated model (IM).

The proposed integration (see Fig 1) of both LWR [11, 12] and Rakha [13] models constitutes a simulation model that emulates the real-time performance measurement [8–9]. This sub-section provides justification on the integration of Rakha and LWR models. The notion to integrate both models is based on the following:

- The optimal behaviour of vehicle dynamics under uninterrupted flow such as speed and acceleration is depicted by the lead vehicle in the platoon. These speed and acceleration characteristics of the lead vehicle can be simulated via the Rakha model.
- Utilizing the optimal speed and travel time characteristics of the lead vehicle as input to the different segments of the LWR model, the arrival profile and interaction of subsequent vehicles at different segments of a link can be modelled based on the three basic postulates of the LWR theory.
- The LWR model has been reported to be able to describe platoon dispersion in [3]. The first vehicle departs from the stop line and move at free flow speed. However, due to varying density and flow in the different segments over time, the subsequent vehicles depart from the stop line and move downstream at a lower speed. The level of dispersion in speeds and, as a result the dispersion in platoon depends on the speed and acceleration characteristics of the lead vehicle.

A few restrictions need to be mentioned on the scope of the application of this model. The following are some of the restrictions:

- The model is applicable to uninterrupted coordinated control arterials which are usually below 1 mile in length. Under uninterrupted conditions, platoons are assumed to progress downstream rather “smoothly” without splitting (due to a slow vehicle in the middle) compared to platoons moving in freeways.
- The model does not take into consideration vehicles overtaking each other and vehicles entering the platoon from the side streets. This is due to the fact that vehicles overtaking are rather minimal in arterials as compared to freeways.

**Fig 2:**
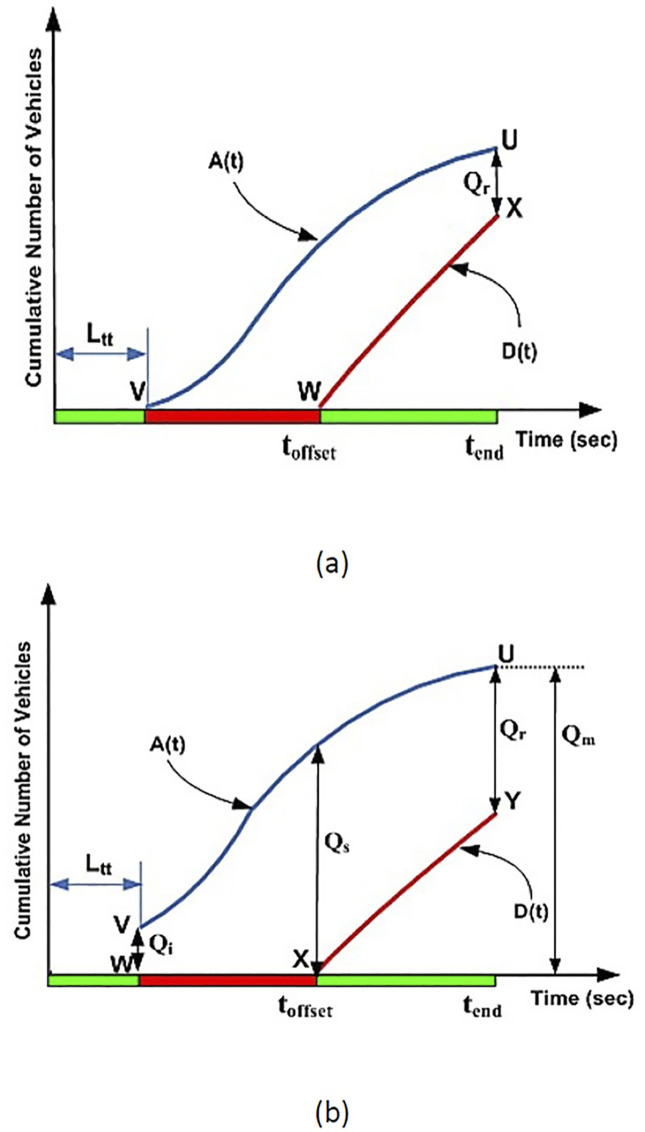
Input-output analytical technique.

The diagram in Fig 1 is described as follows:

- The speed versus time curve of the lead vehicle which is generated by the Rakha model is used to define the maximum speed that a vehicle can attain in each segments of the roadway until it reaches the link free flow speed. Segments before a vehicle reaches free flow speed are 100 meters each whereas the length of the segment where most vehicles are traveling at free flow speed is the remaining distance until the downstream stop line. The ideal speed curve denotes the limited maximal speed *v*_*free i*_ in each LWR-VCPN segments that build on continuum flow.
- The interaction and movement of subsequent vehicles in each segment are “constrained” by the conservation law and the fundamental relationship between flow, speed and density of the LWR-VCPN model.
- Rakha model also estimates the ideal link travel time *L_tt_* of the lead vehicle as it traverses down a motorway from an upstream stop line towards downstream stop line. This is illustrated in the distance versus time curve. *L_ttA_* in the diagram denotes the travel time the lead vehicle takes to reach free flow speed of the motorway whereas *L_ttB_* is the travel time needed for the lead vehicle to reach downstream stop line.
- Arrival curve of vehicles can be obtained in the respective segments of the LWR-VCPN model. The first segment produces the input profile of the vehicles at entrance. The arrival curve A_rr_ff_(t) obtained from the LWR-VCPN segment where all vehicles are assumed to have attained free flow speed describe behaviours of subsequent vehicles e.g. the time the second vehicle and the subsequent vehicles reaching this segment of the motorway relative to the *L_tt_* of the lead vehicle.
- According to actuated performance measurement in [8–9], the arrival profile at free flow speed is time-shifted by a time constant which is simply the distance of the advance detector divided by the free flow speed. Hence, it is justifiable to time-shift the arrival curve obtained from the LWR-VCPN at free flow speed using the travel time of the lead vehicle *L_ttB_*.
- The estimator module in Fig 1 is an algorithm developed to time-shift the arrival profile produced by the LWR-VCPN according to the link travel time *L_ttB_* of the lead vehicle. A typical cumulative platoon arrival profile is illustrated by A_rr_shifted_(t) in the diagram. The platoon width is denoted by clr_*PT*_ which defines the time needed for the platoon to clear the stop line.
- Traffic entering and leaving the motorway is determined by the signal timing plan e.g. phase change data for both upstream and downstream signal. The estimator utilizes the phase change data and downstream saturation flow rate to estimate a departure profile. Combining both arrived A_rr_shifted_(t) and departed vehicles, queues and delays can be estimated using the input and output analytical technique. This is best illustrated in Fig 2.

Fig 2 shows the input-output analysis method. A typical time-shifted arrival profile A(t) obtained from the VCPN is shown in Fig 2(A). On the other hand, a departure profile D(t) can be plotted by the estimator by utilizing the phase change data e.g. the offset between upstream and downstream green as well as the fixed-time green period and cycle time of the downstream signal. Vehicles are assumed to depart from the downstream stop line at saturation flow. Hence, the departure profile undertakes a linear behavior as shown in Fig 2(A). Q_r_ is the residue queue at the ending period of the green signal.

As traffic performance is evaluated from a cycle to cycle basis, Q_r_ from the previous cycle is considered as initial queue Q_i_ for analysis in the subsequent cycle. This is shown in Fig 2(B) where the subsequent arrival profile A(t) joined the initial queue Q_i_. Q_s_ denotes the end of red queue whereas Q_m_ depicts the position of the back of the queue at its peak during the cycle. Calculation of Q_s_ and Q_r_ is denoted by equations (32) and (33) respectively where *t_offset_* is offset time between signals and *t_end_* is the ending time of the approach intersection green period. The total delay encountered by the stopping vehicles is determined by calculating the enclosed area between the arrival and departure profile. This is described by the enclosed area UVWX and UVWXY respectively. Finally, the average delay is given by (34).


$${\text{Q}}_{\text{s}}=\text{A}\left(t_{\mathrm{offset}}\right)-\text{D}\left(t_{\mathrm{offset}}\right)$$ (32)

$${\text{Q}}_{\text{r}}=\text{A}\left(t_{\mathrm{end}}\right)-\text{D}\left(t_{\mathrm{end}}\right)$$ (33)

$$\text{Average delay}=\frac{\text{Total delay}}{\text{Total number of vehicles}}$$ (34)

## Model Application and Implementation

**Fig 3:**
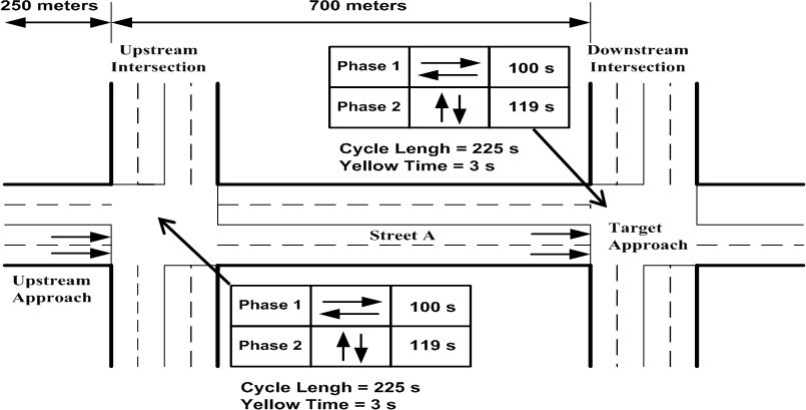
Test intersection.

A pair of fixed-timed intersections (see Fig 3) is used in this paper to simulate queues and average delays from the IM TRANSYT, CTM and HCM2000. The upstream intersection is located 700 m from the target intersection. Both intersections had the same cycle length of 225 s. The upstream intersection assumed an effective green time of 100 s and the cross street green is 119 s. Yellow phase is 3 s. The target intersection also adopts similar signal settings.

Both intersections had “ideal” conditions where only passenger cars are considered, no parking, no transit interference, and no pedestrians. Both intersections had two lanes on each approach that allow through movement only. Right turning and left turning movements are not considered. Traffic volume varied on Street A approaches, however, traffic volume did not vary on Street B and C. Both Street B and C were under-saturated throughout the simulation period. All links had free flow speed of approximately 82 km/h. The upstream intersection has saturation flow of 1980 veh/h/lane whereas the target intersection assumed saturation flow rate of 1800 veh/h/lane.

### 5.1. Model application

In this subsection, we present the application of the IM to describe the test intersection in Fig 3. The link model of the IM is applied to model traffic dynamics along Street A which has length of 700 m. Firstly, speed and travel time characteristics are obtained from the Rakha model using MATLAB. Implementation of the model is based on parameters from a database of thirteen passenger cars compiled by Snare [23]. Using a time step Δt = 1 s, the speeds and distance travelled by a vehicle starting from rest are obtained when equation (12) to (21) are implemented via MATLAB.

**Fig 4:**
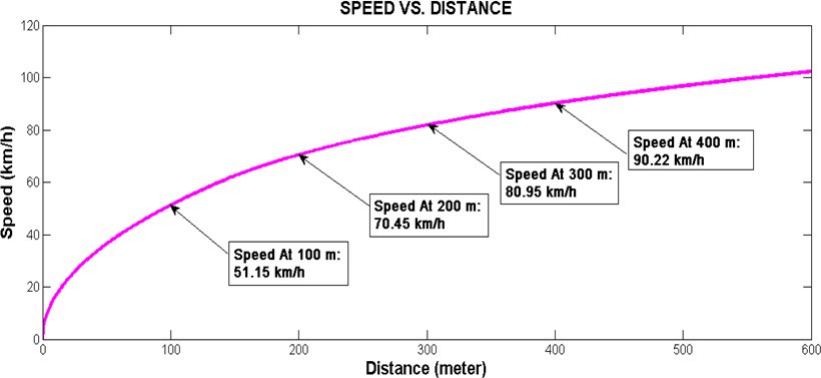
Speed versus distance plot.

**Fig 5:**
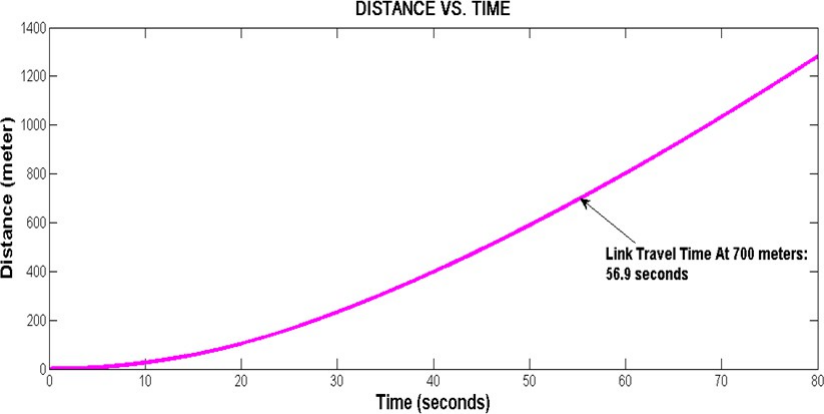
Distance versus time plot.

Simulation of speed versus distance is shown in Fig 4 whereas Fig 5 shows the changes in distance versus time. Referring to Fig 4, it is observed that a vehicle attained speed of 51.15 km/h after it travels 100 m from the upstream stop line; speed of 70.45 km/h, 81.95 km/h and 90.22 km/h are attained at 200 m, 300 m and 400 m respectively. These speeds characteristics depict the limited maximal speed *v*_*free i*_ in each LWR-VCPN segments. As mentioned, the free flow speed of the link is approximately 82 km/h. According to the graph plot in Fig 4, a free flow speed of 81.95 km/h is attained at 300 m downstream. From this point, the vehicle is assumed to continue to move beyond 300 m with the same speed.

On the other hand, Fig 5 depicts the graph of distance versus time. By inspection of the graph, the lead vehicle is expected to reach the downstream stop line of the 700 m motorway in 56.9 seconds. This link travel time *L_tt_* is utilized to time-shift the platoon arrival profile at the target intersection.

**Fig 6:**
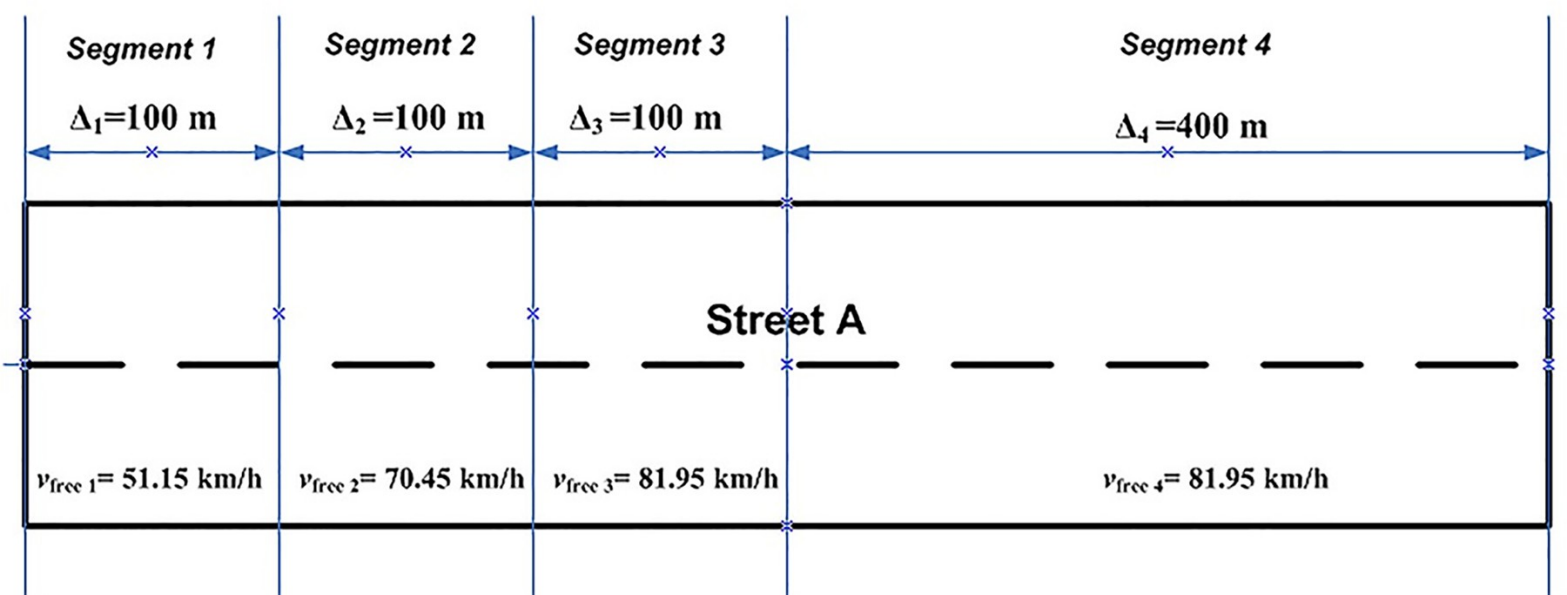
Street A of the test intersection.

**Fig 7:**
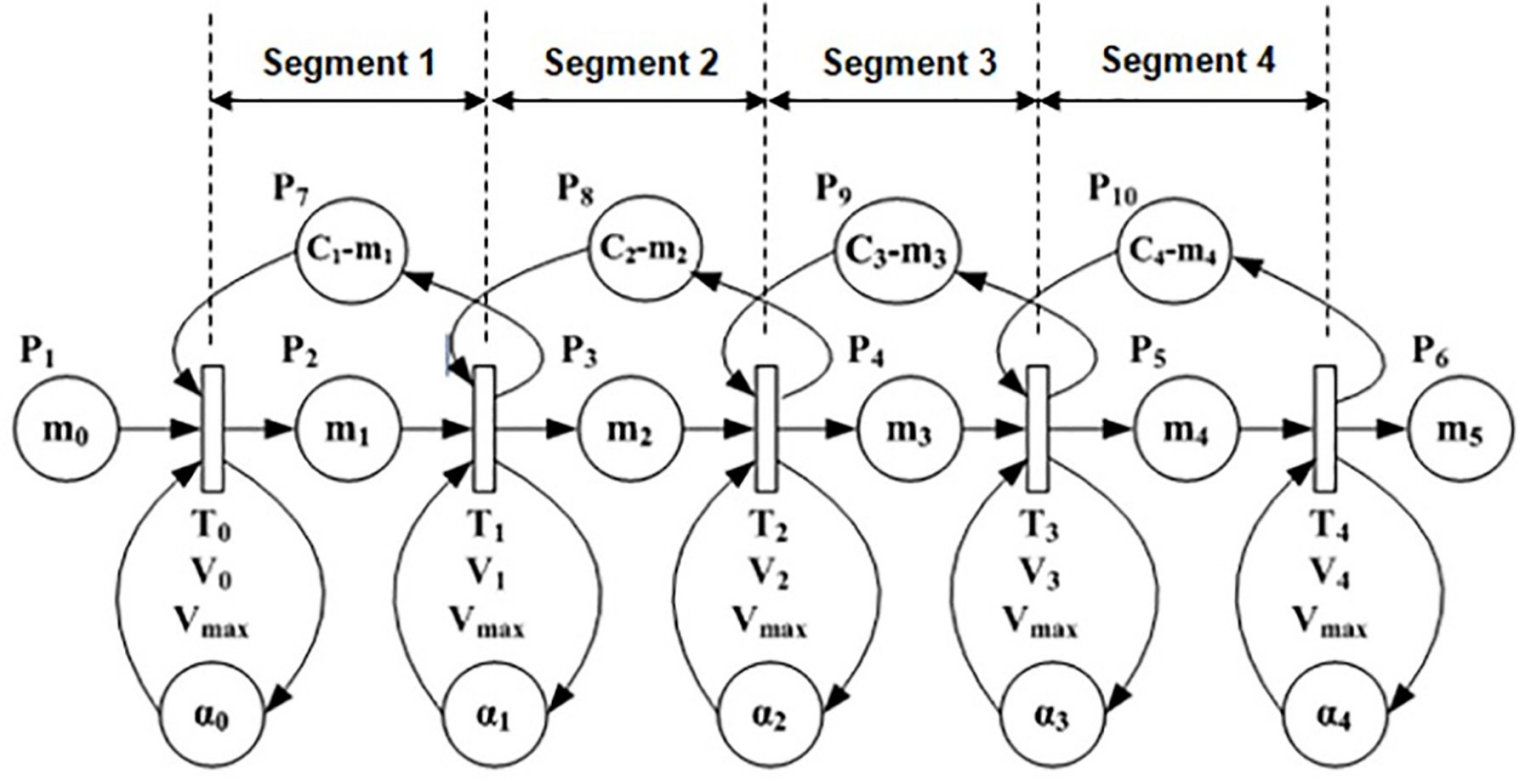
VCPN traffic model. Adapted from models previously developed by Tolba et al. (see Fig 6 of [17] and Fig 9 of [18]).

Street A of the test intersection can be segmentized into four segments as shown in Fig 6 by using speeds profile from the Rakha model. The first three segments have length of 100 m each with their respective limited maximal speed until the vehicle reaches the free flow speed at 300 m. The fourth segment has length of 400 m but vehicles are considered to move in this segment at the free flow speed attained at 300 m. Based on the LWR-VCPN theory by Tolba et al. [17], the authors developed a four-segment VCPN (see Fig 7) to describe platoon dynamics in Fig 6. Marking m_0_ represents the number of vehicles ready to depart from upstream intersection; m_1_, m_2_, m_3_ and m_4_ represent the number of vehicles in segment 1, 2, 3 and 4 respectively; and m_5_ represents the number of vehicles that reached the downstream stop line. Markings C_1_-m_1_, C_2_-m_2_, C_3_-m_3_ and C_4_-m_4_ each represent the number of available sites or available capacity in each segment.

Table 1 outlines the parameters of the VCPN model. Road segments 1, 2 and 3 have equal length of 100 m each whereas segment 4 assumes the remaining length of 400 m. The limited maximal speed *v*_*free i*_ of each segment was obtained from the Rakha model (converted to m/s). All maximal firing frequencies are calculated from the limited maximal speeds using equation (31). The model has a maximal firing frequency V_max0_ which is not listed in Table 1. V_max0_ depicts the firing frequency of vehicles crossing the upstream stop line. Considering saturation flow rate of 1980 veh/h/lane, upstream intersection saturation headway is 1.82 s per vehicle when the signal turns green. V_max0_ is a reciprocal of this saturation headway which is 0.55. The number of available sites C_*i*_-m_*i*_ in each segment is calculated based on the segment length and the maximum occupancy length of 6.6 m per vehicle. Marking invariant *α_i_*(*t*) is calculated based on the maximal flow rate, segment length and the segment limited maximal firing speed (refer to [17]).

**Table 1:** Traffic parameters of VCPN.

Segment	1	2	3	4
Δ_i_(m)	100	100	100	400
*v_free i_*(m/s)	14.21	19.57	22.76	22.76
V_max *i*_(1/s)	0.1421	0.1957	0.2276	0.0569
C_i_-m_i_(veh)	15.15	15.15	15.15	60.61
*α_i_*(*t*) (veh)	3.78	3.78	3.78	15.15

Thus, the VCPN model is ready to be implemented to estimate the arrival profile at Street A target approach. Consequently, the estimator manipulates this arrival profile to calculate queues and average delays. Simulation procedures are explained in the following subsection.

### 5.2. Implementation procedures

The capability of the IM in predicting queues and average delays is evaluated via comparison with the TRANSYT, CTM and HCM2000. Five different traffic demands with differing degree of saturation (DOS) for both under-saturated (*X* = 0.58, 0.7 and 0.86) and oversaturated situations (*X* = 1.1 and 1.2) at the target intersection are studied. Queues and average delays are also simulated considering four arrival types (AT) namely AT-1, AT-2, AT-4 and AT-5 at the target intersection. Hence, 20 different scenarios (four arrival types and five DOS) are simulated.

**Fig 8:**
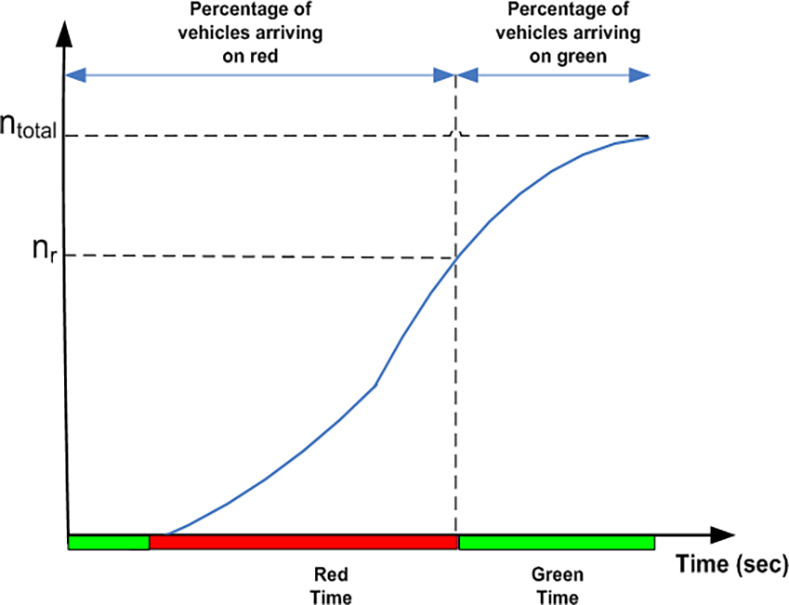
Progression quality.

The IM simulates queues and average delays according to equations (32)–(34). In order to implement simulations under the various scenarios mentioned, offsets between upstream and downstream greens are adjusted to obtain four different arrival types. By varying the offset between upstream and downstream greens, the quality of progression of a platoon of vehicles at the target approach varies. Arrival types could then be ascertained by assessing the quality of progression i.e. the percentage of vehicles arriving during the red or green period. Fig 8 shows a typical arrival profile from the IM. The percentage of vehicles reaching during red can be calculated using equation (35) whereas percentage of vehicles reaching during the green period is determined by (36). Therefore, suitable offsets are used to produce AT-1, AT-2, AT-4 and AT-5 arrivals.

Percentage of vehicles arriving during the red phase:

$$\frac{n_{r}}{n_{\mathrm{total}}}\times 100\%$$ (35)

Percentage of vehicles arriving during the green phase:

$$100-\left(\frac{n_{r}}{n_{\mathrm{total}}}\times 100\%\right)$$ (36)

where *n_total_* and *n_r_* denote the total number of vehicles and the number of vehicles arriving during the red period respectively.

Secondly, the desired DOS can be achieved through ascertaining the number of vehicles entering the VCPN. The capacity of the Street A target approach is 800 veh/h. Based on this capacity, traffic demand (veh/h) to achieve the desired DOS can be calculated. For instance, to achieve *X* = 0.7, entering flow of 560 veh/h is needed (see Table 2). Considering an analysis period of 15 minutes, the total demand of through vehicles approximated from this entering flow is 140. As an analysis period of 15 minutes is considered, simulation needs to be performed for four signal cycles (i.e. four times of 225 s). Thus, the demand for each signal cycle is 35 vehicles. Table 2 outlines all the demands to achieve the respective DOS.

**Table 2:** Traffic demands for the link model (*capacity* = 800 veh/h).

Degree of Saturation (DOS) *X*	Traffic demands To achieve DOS (veh/h)	Traffic demands For 15 minutes (vehs)	Traffic Demands For one signal cycle (vehs)
**0.58**	464	116	29
**0.7**	560	140	35
**0.86**	688	172	43
**1.1**	880	220	55
**1.2**	960	240	60

Demand for one signal cycle denotes the marking of m_0_ in the VCPN model. Utilizing the initial marking of m_0_ (i.e. 29, 35, 43, 55 and 60 vehicles respectively), the IM evaluates traffic from a cycle to cycle basis. The respective initial marking is used to evaluate traffic under AT-1, AT-2, AT-4 and AT-5 arrivals. Thus, all the 20 scenarios mentioned can be simulated with these settings. Estimated arrival profile for each scenario is used by the estimator (which is coded in MATLAB) to simulate Q_s_, Q_m_, Q_r_ and total delay from a cycle to cycle basis. As mentioned, a total of four signal cycles (equivalent to 15 minutes analysis period) is performed for each scenario using the appropriate demands listed in Table 2.

For each scenario simulated, the average delay is obtained by dividing the total delays accumulated over four signal cycles with the demand of through vehicles (vehs) during this period as shown in equation (37). On the other hand, equation (38) shows calculation of the mean maximum queue Q_MM_ which is the estimated mean over all cycles of the position of the back of the queue at its peak. The maximum end of red queue Q_MEOR_ is the highest value of Q_s_ that can be achieved within the analysis period.


$$\text{Average delay}=\frac{\text{Total delay over four signal cycles}}{\text{Demand of through vehicles for}\;15\;\text{minutes}}$$ (37)

$${\text{Q}}_{\text{MM}}=\frac{\text{Sum of}\;{\text{Q}}_{\text{m}}\;\text{over four signal cycles}}{4}$$ (38)

$${\text{Q}}_{\text{MEOR}}=\text{highest value of}\;{\text{Q}}_{\text{s}}\;\text{that can be achieved within the analysis period}$$ (39)

## Comparison of Simulation Results

### 6.1. IM simulations

The link model and the signal timing plan for both intersections are implemented and simulated in the MATLAB environment where an analysis period of 15 minutes is considered. Simulation for AT-1 and DOS of *X* = 0.58 will be used to illustrate the implementation of the IM. According to Table 2, the demand over 15 minutes time period for *X* = 0.58 is 116 vehicles. The link model needs to be implemented for four signal cycles (225 s per cycle) to attain a 15 minutes analysis period. Therefore, for each cycle, 29 vehicles passed through the upstream intersection. Thus, the initial marking of m_0_ in the VCPN is set to this value.

**Fig 9:**
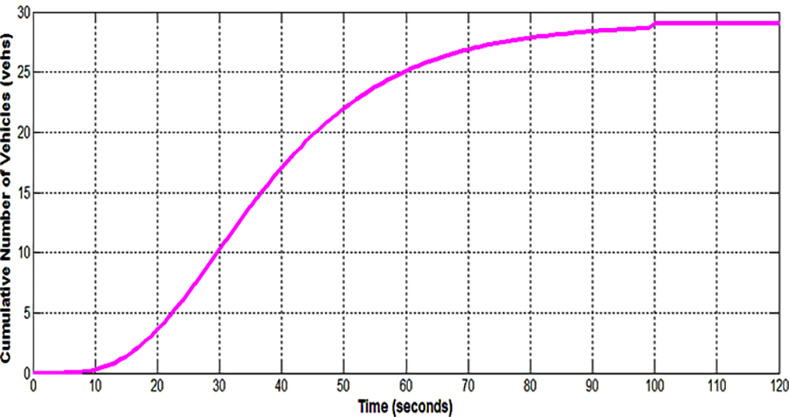
Cumulative platoon arrival profile from LWR-VCPN.

**Fig 10:**
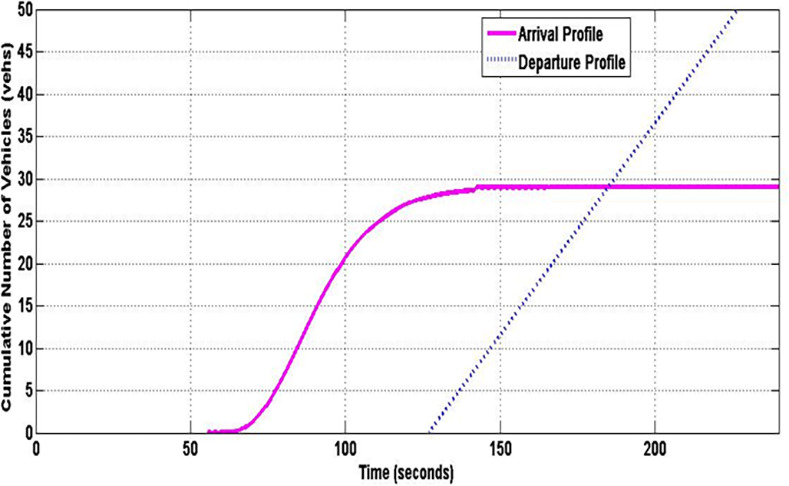
Arrival and departure profile.

Simulated platoon arrival profile at downstream stop line (i.e. place m_5_) from the VCPN for the first cycle of implementation is shown in Fig 9. This arrival profile is time-shifted by the estimator using *L_tt_* = 56.9 s (see Fig 10). Based on the existing timing plan of the target approach and suitable offset between intersections, arrival AT-1 is achieved where approximately 28 vehicles (96.08% of the platoon) arrived during the red period. The estimator plotted the departure profile and calculates Q_s_, Q_m_, Q_r_ and total delay. Any residue queue Q_r_ will be considered as the initial queue for the analysis in the next cycle. However, there is no resultant Q_r_ as all the vehicles cleared the intersection stop line at the end of the green period. The total delay for the implementation for this cycle is 1791.9 seconds by calculating the area encapsulated by the arrival and departure profiles. Similar simulation is run for another three consecutive cycles. Simulated results over four cycles are shown in Table 3.

**Table 3:** Simulated queues and total delays for X = 0.58.

Cycle	Demand for one signal cycle (vehs)	End of red queue Q_s_ (vehs)	Residue queue Q_r_ (vehs)	Peak position of queue Q_m_ (vehs)	Total delay (s)
**1**	29	28	0	29	1791.9
**2**	29	28	0	29	1791.9
**3**	29	28	0	29	1791.9
**4**	29	28	0	29	1791.9
**Total**	116	-	-	116	7167.6

As observed in Table 3, overall total delay for the 15 minutes period is 7167.6 s. Division of this value by 116 gives an average delay 61.79 s/veh. The value of Q_MEOR_ is 28 whereas Q_MM_ is 29 when the overall total of Q_m_ is divided by 4. These procedures are repeated for the other 19 scenarios. Simulated queues and average delays for all scenarios are summarized and compared with TRANSYT, CTM and HCM2000 results in section 6.5.

### 6.2. TRANSYT simulations

**Fig 11:**
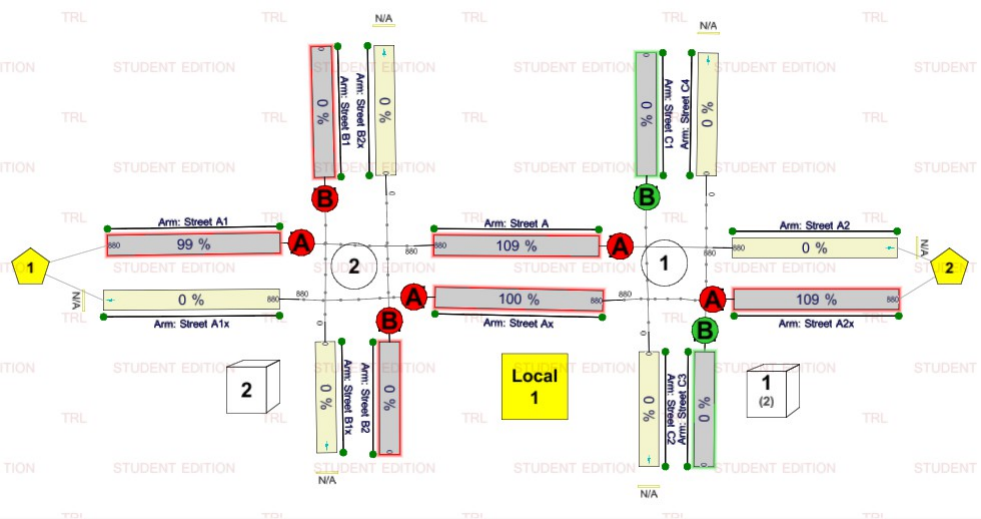
TRANSYST model of the test intersection.

The test intersection in Fig 3 is modeled with TRANSYT version 14 (See Fig 11). The simulation employed a platoon dispersion model (PDM) with analysis period set to 15 minutes. The following are some procedures to run the model according to the scenarios mentioned:

- The model lane lengths are set according to the specifications in Fig 3. Free flow speed on street A is set to 82 km/h. The saturation flow of upstream traffic is set to 1980 veh/h/lane whereas downstream approach is set to 1800 veh/h/lane.
- To achieve the respective DOS of 0.58, 0.7, 0.86, 1.1 and 1.2; origin-destination flow from node 1 to node 2 (viz. the demand flow rate along Street A) is set to 464, 560, 688, 880 and 960 veh/h respectively.
- Offset between upstream signal phase A (phase 1) and the target approach phase A (phase 1) is adjusted to achieve the desired arrival types AT-1, AT-2, AT-4 and AT-5 for each of the demand flow rate. Hence, all 20 scenarios can be simulated.
- Results of Q_MM_, Q_MEOR_ and average delays for each scenario can be obtained from the TRANSYT report viewer.

### 6.3. HCM 2000 Computations

The test intersection has a cycle length *C* = 225s; effective green time *g* = 100s; *u* = *g/C* = 0.44; saturation flow rate *s* = 1800 veh/h; analysis period *T* = 0.25 h; *k* = 0.5 for pre-timed signal and capacity *c* = 800 veh/h. The following computations are conducted:

- *PF* values are calculated based on default values of *P* and *f*_PA_ in the HCM2000. The calculated *PF* values for each arrival type AT-1, AT-2, AT-4 and AT-5 are 1.53, 1.18, 0.84 and 0.47 respectively.
- *I* values are subjected to the degree of saturation of an upstream intersection (*X_u_*) as follows:
$$I=1.0-0.91X_{u}^{2.68}\;X_{u}\leq 1$$ (40)
  As there is a similar intersection upstream, *X_u_* is assumed to be the same as downstream DOS (*X*). Consequently, the *I* values corresponding to the *X_u_* of 0.58, 0.7, 0.86, 1.1 and 1.2 are 0.789, 0.650, 0.383, 0.09 and 0.09 respectively.
- Calculation of average delays considers *d_3_* = 0 since there is no initial queue considered in the analysis period. Thus, the equation for average delay calculation in equation (5) takes the following form.
-
$$d=d_{1}\left(PF\right)+d_{2}$$ (41)
  The given constant values of *C, u, T, c* and *k* are applied directly into the delay equation (6) and (7). Subsequently, average delays for each scenario can be calculated using appropriate values of *PF, I* and *X*.
- Calculation of the mean maximum queue Q_MM_ is similar to calculation of delays. The demand flow rate *V* that produces the respective DOS(*X*) are 464, 560, 688, 880 and 960 veh/h respectively. By utilizing appropriate values of *PF_2_*, *X, V* and *k_B_* and the constant values of *C, u, T, c* and *s* into equation (9); the mean maximum queue Q_MM_ can be approximated for all the scenarios mentioned.

### 6.4. CTM Simulations

CTM is implemented on street A using the equations outlined in section 2.2. Equations (2), (3) and (4) are implemented using an excel spreadsheet. The parameters for the CTM are set as follows:

- The length of each clock tick: 5 s
- Jam density: 105 veh/km (i.e. 6.6 m for every vehicle)
- Free-flow speed: 82 km/h (i.e. 22.78 m/s)
- Flow capacity: 1800 veh/h/lane (i.e. *Q* = 2.5 vehicles/time interval/lane)
- Number of cells for Street A: 6 cells (i.e. the length of Street A is 700 m)
- The holding capacity of each cell is *N* = 11.96 vehicles.

Similar to the implementation of the IM, simulation is conducted for four signal cycles to achieve an analysis period of 15 minutes. The respective DOS of 0.58, 0.7, 0.86, 1.1 and 1.2 are achieved using entry volume of 29, 35, 43, 55 and 60 vehicles for each signal cycle. Offset between upstream signal and the target approach is adjusted to achieve the desired arrival types AT-1, AT-2, AT-4 and AT-5 for each of the demand flow rate. Hence, all 20 scenarios can be simulated.

- Q_MEOR_ is the maximum end of red queue that can be obtained throughout the analysis period.
- The delay at the cell level $d_{a}^{i}(t)$ is given by equation (42). $n_{a}^{i}(t)$ is the number of vehicles occupying cell *i* and $y_{a}^{i+1}(t)$ is the outflow of the cell. It follows that if the exit flow from cell *i* at time interval *t* is less than its current occupancy due to congestion, then the vehicles which cannot leave the cell will incur a delay of one time step. Once the delay has been determined at the cell level, the total delay at the network level can be ascertained using equation (43). Average delay is obtained by dividing total delay with the total vehicles entering the network during the analysis period.

$$d_{a}^{i}\left(t\right)=n_{a}^{i}\left(t\right)-y_{a}^{i+1}\left(t\right)$$ (42)

$$d\left(t\right)=\Sigma_{a}\Sigma_{i}d_{a}^{i}\left(t\right)$$ (43)

### 6.5. Comparison of queues and average delays

Simulated results of queues and average delays for all 20 scenarios from IM, TRANSYT, CTM and HCM2000 are compared for both under-saturated and oversaturated situations. Three performance indices namely the maximum end of red queue Q_MEOR_, mean maximum queue Q_MM_ and average delays are compared to assess the feasibility of the IM.

Table 4 shows a comparison of Q_MEOR_. However, only Q_MEOR_ from IM, TRANSYT (notated as TSYT in the respective tables) and CTM are compared as HCM2000 does not have any provision for calculation of Q_MEOR_. Table 5 shows comparison of Q_MM_ between IM, TRANSYT and HCM2000 (notated as HCM in the respective tables) as CTM did not provide avenue for estimation of Q_MM._ Table 6 displays the overall average delays simulated from all the models.

**Table 4:** Comparison of maximum end of red queues Q_MEOR_ (T = 15 min).

Arrival Type	DOS *X*	TYST (vehs)	IM (vehs)	CTM (vehs)
**AT-1**	0.58	28.38	27.86	32.50
	0.7	34.53	33.62	32.50
	0.86	43.51	41.68	39.39
	1.1	61.38	67.83	55.00
	1.2	69.96	87.61	60.00
**AT-2**	0.58	22.83	22.84	27.50
	0.7	28.24	27.35	27.50
	0.86	35.48	33.94	30.00
	1.1	54.81	58.32	54.27
	1.2	63.38	77.29	53.62
**AT-4**	0.58	15.56	16.82	10.00
	0.7	20.81	20.53	15.00
	0.86	26.48	25.39	17.50
	1.1	43.70	47.47	43.91
	1.2	52.28	54.42	44.28
**AT-5**	0.58	4.20	5.66	5.00
	0.7	5.43	6.76	5.00
	0.86	8.10	8.51	8.00
	1.1	22.87	25.54	22.50
	1.2	31.44	41.58	28.56

**Table 5:** Comparison of mean maximum queues Q_MM_ (T = 15 min).

Arrival Type	DOS *X*	TSYT (vehs)	IM (vehs)	HCM (vehs)
**AT-1**	0.58	29.38	29.00	27.06
	0.7	35.75	35.00	37.10
	0.86	45.05	43.00	49.42
	1.1	63.19	62.50	79.34
	1.2	71.77	72.50	99.02
**AT-2**	0.58	29.21	29.00	27.06
	0.7	35.71	35.00	34.91
	0.86	45.04	43.00	48.05
	1.1	63.19	62.50	72.96
	1.2	71.77	72.50	83.00
**AT-4**	0.58	26.60	29.00	20.43
	0.7	35.43	35.00	28.21
	0.86	45.02	43.00	43.17
	1.1	63.19	62.50	79.34
	1.2	71.77	72.50	99.02
**AT-5**	0.58	22.43	29.00	15.60
	0.7	30.07	35.00	22.68
	0.86	43.37	43.00	38.15
	1.1	63.16	62.50	91.00
	1.2	71.75	72.50	145.68

**Table 6:** Comparison of Average Delays (T = 15 min).

Arrival Type	DOS *X*	TYST (s/veh)	IM (s/veh)	CTM (s/veh)	HCM (s/veh)
**AT-1**	0.58	92.21	61.79	62.15	74.16
	0.7	97.06	66.64	86.06	80.63
	0.86	106.24	79.18	98.46	91.22
	1.1	140.89	123.01	116.38	142.98
	1.2	163.83	147.83	117.63	187.05
**AT-2**	0.58	51.27	36.01	41.74	57.52
	0.7	60.07	42.73	60.37	62.69
	0.86	74.25	51.31	62.95	71.22
	1.1	128.95	100.27	114.53	120.74
	1.2	150.37	128.70	116.20	164.81
**AT-4**	0.58	27.25	23.85	25.00	41.89
	0.7	40.19	29.19	30.00	45.85
	0.86	57.27	37.38	35.00	52.44
	1.1	108.76	88.41	95.84	99.82
	1.2	131.86	107.83	113.36	143.93
**AT-5**	0.58	9.45	16.69	25.00	24.24
	0.7	13.54	22.19	30.00	26.84
	0.86	23.64	27.75	31.12	31.24
	1.1	70.93	67.70	42.21	76.28
	1.2	97.18	94.82	53.77	120.35

The maximum end of red queue Q_MEOR_ (as shown in Table 4) for IM, TRANSYT and CTM are found to be in closer agreement under under-saturated situation (*X* = 0.58, 0.7 and 0.86). IM tends to estimate higher queues for oversaturated conditions compared to TRANSYT and CTM. This is most obvious when *X* = 1.2 for all arrival types. In TRANSYT, queue lengths are derived from cyclic flow profiles during each step of the typical cycle. ‘Uniform’ component of queue is derived from the cyclic flow profiles. Additional elements associated with random and oversaturated effects are added to it to derive queues for oversaturated situation. On the other hand, IM calculates both under-saturated and oversaturated queues directly from the arrived and departed profiles based on the input-output theory.

Table 5 shows that Q_MM_ from TRANSYT and IM closely agree for all scenarios. The HCM2000 estimation have closer values with Q_MM_ from TRANSYT and IM for most under-saturated situations (*X* = 0.58, 0.7 and 0.86). However, Q_MM_ computed by the HCM2000 increased drastically when traffic becomes oversaturated.

**Fig 12:**
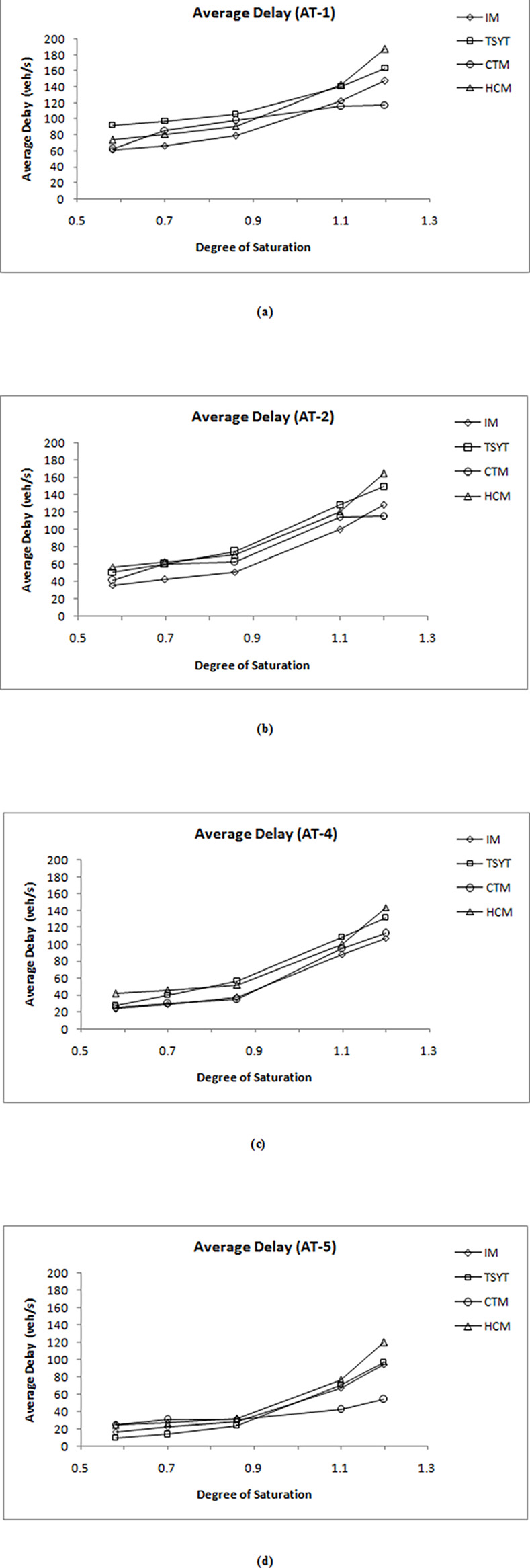
Comparison of average delays for all scenarios.

Comparison of average delays shown in Table 6 are plotted into graph plots of average delays versus degree of saturation for all 20 scenarios (see Fig 12(A) to 12(D)). Both TRANSYT and HCM2000 are found to produce higher delays compared to IM for all DOS under AT-1, AT-2 and AT-4 arrivals. However, the average delays of TRANSYT are closer in agreement with IM for all DOS under AT-5 arrival. HCM2000 computes the highest value of delay compared to the other three models for *X* = 1.2 under all arrival types. CTM average delays closely agrees with IM for *X* = 0.58 and *X* = 1.1 under AT-1 arrival. Average delays for AT-2 and AT-4 arrivals simulated by CTM seemed to agree closely with IM for most DOS. The average delays by CTM are closer in agreement with IM for under-saturated situations under AT-5 arrival but started to ‘deteriorate’ when traffic became oversaturated.

**Fig 13:**
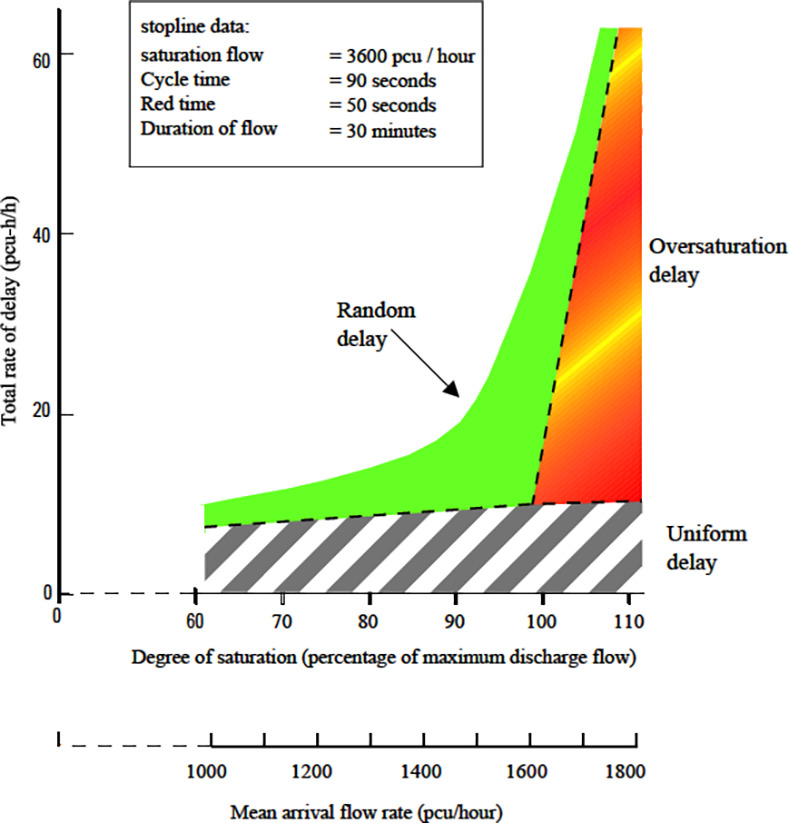
Traffic delay on a link.

The simulation results are analyzed as follows:

1. Both Q_MEOR_ and Q_MM_ simulated by IM closely agree with TRANSYT. Though IM estimated higher queues especially at *X* = 1.2 for all arrival types, this is primarily caused by the differences in the method of calculating queues for oversaturated situation. Despite the variation found in oversaturated situation, the ‘uniform’ component of queues is observed to agree closely for both IM and TRANSYT. On the other hand, comparison of Q_MM_ produced by HCM2000 yielded close agreement for under-saturated traffic. HCM2000 tends to estimate higher queues for oversaturated situations compared to IM and even TRANSYT. This ‘phenomenon’ of high estimation of oversaturated queues by HCM2000 had also been reported in [24].
2. Average delays simulated by the IM are much lower compared to TRANSYT and HCM2000 for most scenarios. Both HCM2000 and TRANSYT adopt similar method for average delay calculation. Hence, average delays from HCM2000 and TRANSYT seemed to coincide. The calculation of average delay in the IM is based on the area enclosed by the arrival and departure profile (see Fig 2) where else average delays in TRANSYT and HCM2000 are calculated using analytical expression considering three components of delays namely uniform, random and oversaturated delays (see Fig 13). The discrepancies of the IM delays compared to TRANSYT and HCM2000 may be due to the fact that these mathematical expressions tend to overestimate the random component of delay, particularly for links that are well below capacity [25]. Fig 13 illustrated an increase in the degree of saturation from 95 to 100 per cent will increase random delay by some 80 per cent in TRANSYT. In addition, this overestimation of delays by HCM2000 was also reported in [26] and [27].
3. Average delays from CTM and IM agree closely for most DOS under AT-2 and AT-4 arrivals. Another observation reveals that CTM delays agree quite closely with TRANSYT for all under-saturated situations for all arrival types. However, CTM estimation for oversaturated situation did not reveal a substantial increase and could not ‘catch up’ with TRANSYT and HCM2000. Hence, it tends to underestimates delay for good progression (e.g. AT-5) when traffic became oversaturated. CTM also simulated average delays that are lower compared to IM when traffic became oversaturated for AT-1, AT-2 and AT-5 arrivals. Performance of CTM is also reported by Feldman and Maher [28] to be comparable with TRANSYT in circumstances where no blocking back is to be expected. However, when a blocking back occurs, the two models showed a substantial discrepancy in their performances. This explains the tendency of CTM to predict lower delays when traffic became oversaturated i.e. when blocking back occurs. This may also be due to the CTM inability to model platoon dispersion appropriately [29].

## Conclusions

To this end, we have presented an integrated model (IM) that comprises of Rakha and LWR models integrated into VCPN to describe platoon arrivals and evaluate performance of signalized arterials. An estimator is developed to manipulate the predicted arrival profiles and perform calculations of queues and average delays. This research has shown that the IM can describe reasonably platoon arrivals in arterial by simulating platoon cumulative arrival profiles and traffic parameters that are utilizable. This enables platoon progression to be approximated fairly well at a signalized intersection. This is proven as queues derived from ‘uniform’ arrivals for both TRANSYT and IM closely agree. In other words, profile generated by IM is similar to the cyclic profiles in TRANSYT. Simulated average delays yielded acceptable disparity which has been appropriately analyzed and explained. Considering the related factors that contributed to these disparities, the IM has been somewhat reasonably validated.

As a final emphasis, the IM aims to bring about the following benefits:

- Analysis of the arrival profile from the LWR using input-output technique is less tedious than shockwave analysis. The model application has shown that the LWR model can be analyzed using this technique without considering the effect of shockwave.
- The IM also shows the integration with the Rakha vehicle dynamics model provide the LWR-VCPN with useful information which is similar to the fundamental diagram commonly used in most shockwave tracking models. In addition, the IM can be implemented with lesser number of *cells* compared to the CTM. This directly implies lesser computational time.
- The IM is able to produce platoon profile that could be used to estimate the expected arrival (departure) time of the first (last) vehicle in a particular platoon which could not be provided by shockwave tracking models due to their inability to model platoon dispersion appropriately. These estimates from the IM are particularly useful in self-scheduling control approach that relies on these time parameters of the platoon to regulate signal timing plans and optimize traffic progression through the intersection.
- TRANSYT and HCM2000 analyze and produce traffic performances over a period of time called the analysis period. Hence, traffic inputs and simulation results are aggregated values in these models. However, the IM provides the facility to predict traffic performances from a cycle to cycle basis. Hence, it provides a more accurate representation of the traffic from a cycle to cycle basis.

Finally, the authors suggest further work to be conducted to validate the IM with data collected from real-world traffic intersection. This will further ascertain the viability of the model.

## Supporting information

S1 File CTM delay and maximum end of red queue computations for DOS 0.7. (XLSX)

S2 File CTM delay and maximum end of red queue computations for DOS 0.58. (XLSX)

S3 File CTM delay and maximum end of red queue computations for DOS 0.86. (XLSX)

S4 File CTM delay and maximum end of red queue computations for DOS 1.1. (XLSX)

S5 File CTM delay and maximum end of red queue computations for DOS 1.2. (XLSX)

S6 File HCM delay and mean maximum queue computations. (XLSX)
