# Correction: An Integrated Approach for Platoon-based Simulation and Its Feasibility Assessment

**DOI:** 10.1371/journal.pone.0306215

**Published:** 2024-07-09

**Authors:** Kok Mun Ng, Mamun Bin Ibne Reaz

## Notice of republication

After publication of this article [1], concerns were raised about similarities of this work to previously published articles [2, 3, 4], reuse of equations and figures from those articles, and whether these related works were sufficiently discussed in the article so as to place the new work in the *PLOS ONE* article [1] into context.

To address these issues, the article [1] was republished at the time of this notice’s publication to incorporate the following changes:

- The Introduction has been updated to explain how this article builds upon previous work.
- The Abstract and LWR-IM Model sections in the original published version indicated that this work was based on previous work [4], but the article [1] did not clearly discuss that the two main components of the LWR-IM model, namely the Rakha vehicle dynamics and VCPN models, were published previously. The Theoretical Background section of [1] has been updated to indicate where the article discusses equations and theory previously published by Rakha et al. (ref. 13 in [1]) and Tolba et al. (ref. 17–18 in [1]).
- Figs 1 and 2,in the original published version of [1] reproduced content previously published in [2, 3]. The original sources were not cited, and the authors did not have permission to publish the figures in *PLOS ONE* under a CC BY license. Figs 1 and 2 are removed in the updated version, and the text now refers readers to the original publications.
- Figs 8 and 9 in the original published version of [1] were similar to content published previously in [2, 3]. In the updated version of [1], these figures have been revised and renumbered as Figs 6 and 7; the legend of the new Fig 7 specifies the model was adapted from those previously published in [2, 3], and an in-text citation was added attributing the underlying LWR-VCPN theory to Tolba et al. (ref. 17 in [1]).
- The figures have been renumbered in the republished article to account for the above changes.

The *PLOS ONE* Editors consider that the above amendments resolve the concerns regarding attribution.

The primary data for this study were not included with the article, contrary to the Data Availability statement. At time of republication the original article was updated to include the raw and supplementary data for CTM and HCM 2000 in S1-S6 Files. The results for the IM were captured at the MATLAB work space at the point of simulation; hence, it was already tabulated into the tables in the article. The results for TRANSYT were also captured at the point of simulation and were already tabulated into the tables. The TRANSYT software is a student package that expired in 2015.

Please download this article again to view the correct version.
